# The Hippo kinases control inflammatory Hippo signaling and restrict bacterial infection in phagocytes

**DOI:** 10.1128/mbio.03429-23

**Published:** 2024-04-16

**Authors:** Brendyn M. St. Louis, Sydney M. Quagliato, Yu-Ting Su, Gregory Dyson, Pei-Chung Lee

**Affiliations:** 1 Department of Biological Sciences, College of Liberal Arts and Sciences, Wayne State University, Detroit, Michigan, USA; 2 Department of Oncology, School of Medicine, Wayne State University, Detroit, Michigan, USA; University of Michigan-Ann Arbor, Ann Arbor, Michigan, USA; Tufts University School Of Medicine, Boston, Massachusetts, USA

**Keywords:** Stk3, Stk4, KrsA, KrsB, pathogenesis

## Abstract

The Hippo kinases MST1 and MST2 initiate a highly conserved signaling cascade called the Hippo pathway that limits organ size and tumor formation in animals. Intriguingly, pathogens hijack this host pathway during infection, but the role of MST1/2 in innate immune cells against pathogens is unclear. In this report, we generated *Mst1/2* knockout macrophages to investigate the regulatory activities of the Hippo kinases in immunity. Transcriptomic analyses identified differentially expressed genes (DEGs) regulated by MST1/2 that are enriched in biological pathways, such as systemic lupus erythematosus, tuberculosis, and apoptosis. Surprisingly, pharmacological inhibition of the downstream components LATS1/2 in the canonical Hippo pathway did not affect the expression of a set of immune DEGs, suggesting that MST1/2 control these genes *via* alternative inflammatory Hippo signaling. Moreover, MST1/2 may affect immune communication by influencing the release of cytokines, including TNFα, CXCL10, and IL-1ra. Comparative analyses of the single- and double-knockout macrophages revealed that MST1 and MST2 differentially regulate TNFα release and expression of the immune transcription factor MAF, indicating that the two homologous Hippo kinases individually play a unique role in innate immunity. Notably, both MST1 and MST2 can promote apoptotic cell death in macrophages upon stimulation. Lastly, we demonstrate that the Hippo kinases are critical factors in mammalian macrophages and single-cell amoebae to restrict infection by *Legionella pneumophila*, *Escherichia coli*, and *Pseudomonas aeruginosa*. Together, these results uncover non-canonical inflammatory Hippo signaling in macrophages and the evolutionarily conserved role of the Hippo kinases in the anti-microbial defense of eukaryotic hosts.

**IMPORTANCE:**

Identifying host factors involved in susceptibility to infection is fundamental for understanding host-pathogen interactions. Clinically, individuals with mutations in the MST1 gene which encodes one of the Hippo kinases experience recurrent infection. However, the impact of the Hippo kinases on innate immunity remains largely undetermined. This study uses mammalian macrophages and free-living amoebae with single- and double-knockout in the Hippo kinase genes and reveals that the Hippo kinases are the evolutionarily conserved determinants of host defense against microbes. In macrophages, the Hippo kinases MST1 and MST2 control immune activities at multiple levels, including gene expression, immune cell communication, and programmed cell death. Importantly, these activities controlled by MST1 and MST2 in macrophages are independent of the canonical Hippo cascade that is known to limit tissue growth and tumor formation. Together, these findings unveil a unique inflammatory Hippo signaling pathway that plays an essential role in innate immunity.

## INTRODUCTION

The Hippo pathway exists in all eukaryotes from single-cell amoebae to multicellular organisms like humans (1) and controls important biological functions, such as cell cycle progression and organ development (2, 3). In canonical Hippo signaling of mice and humans, mammalian STE20-like protein kinases-1 and -2 (MST1 and MST2) are the Hippo kinases that phosphorylate the scaffold protein, Mps one binder kinase activator-like 1A and B (MOB1A/B), and the downstream kinases, large tumor suppressor homolog 1 and 2 (LATS1/2). Phosphorylated MOB1 and LATS1/2 form active kinase complexes which subsequently phosphorylate the transcription regulators, Yes-associated protein 1 (YAP1), and WW domain-containing transcription regulator protein 1 (WWTR1). Once phosphorylated, YAP1 and WWTR1 are sequestered in the cytoplasm or undergo proteasomal degradation. Conversely, when canonical Hippo signaling is off, unphosphorylated YAP1/WWTR1 shuttle into the nucleus and interact with the transcription enhancer factors TEADs to control gene expression (2–6).

While *Mst1/2* double knockout (*Mst1/2^–/–^*) leads to embryonic lethality (7), conditional *Mst1/2^–/–^* in the liver or intestines causes enlarged tissues and tumor formation in mouse models (8–11). Ectopic overproduction of MST1 or MST2 in cell lines promotes apoptosis (12–16), and elevated YAP1 protein levels are associated with human cancers (17), suggesting that MST1/2 possess pro-apoptotic activities to limit cell proliferation and tumors. However, naïve T cells isolated from *Mst1/2^–/–^* mice and humans with defective MST1 are highly sensitive to apoptosis (18–20), indicating that the regulatory activities of MST1/2 in programmed cell death depend on specific cell types and conditions. While being one of the key pathways in human cancer research (21), the Hippo pathway also has an emerging role in immunity. Humans with loss-of-function mutations in the *MST1* gene have clinical histories of recurrent infections and pneumonia (19, 20, 22). Mice with conditional *Mst1/2^–/–^* in myeloid cells are more susceptible to death caused by septic peritonitis (23). However, it remains undefined if MST1 and MST2 have shared or distinct roles in host defense. Meanwhile, evidence implicating the Hippo pathway’s role in microbial pathogenesis is rising. The opportunistic pathogen *Legionella pneumophila* uses LegK7, a bacterial effector protein, to hijack the Hippo scaffold protein MOB1 (24, 25). Similarly, *Chlamydia trachomatis* and *Salmonella enterica* use the effector proteins Tarp and SopB, respectively, to manipulate the Hippo transcription regulator YAP1 (26, 27). Moreover, human papillomavirus E6 protein targets LATS kinases and TEAD transcription factors to promote pathogenesis (28, 29), and replication of the SARS-CoV2 virus is increased in host cells with MST1/2 knockdown (30).

Here, we use comparative genetic approaches to investigate the role of MST1/2 in macrophages, the professional phagocytes in innate immunity. We found that MST1 and MST2 are critical regulators for the expression of hundreds of macrophage genes involved in immune disorders and cell death pathways. Interestingly, MST1 and MST2 regulate a set of immune genes through inflammatory Hippo signaling cascades that are independent of the canonical Hippo pathway. MST1 and MST2 have different regulatory effects on the release of cytokines in macrophages, indicating that the Hippo kinases affect cell-cell communication in immunity. Lastly, we demonstrate the requirement of MST1/2 in programmed cell death of macrophages and characterize the conserved Hippo kinases as critical host factors in macrophages and protozoans to restrict invading bacterial pathogens. These findings reveal an inflammatory Hippo signaling pathway in innate immunity that is unique and distinct from the canonical Hippo pathway in organ development.

## RESULTS

### The macrophage transcriptome controlled by MST1/2

**Fig 1:**
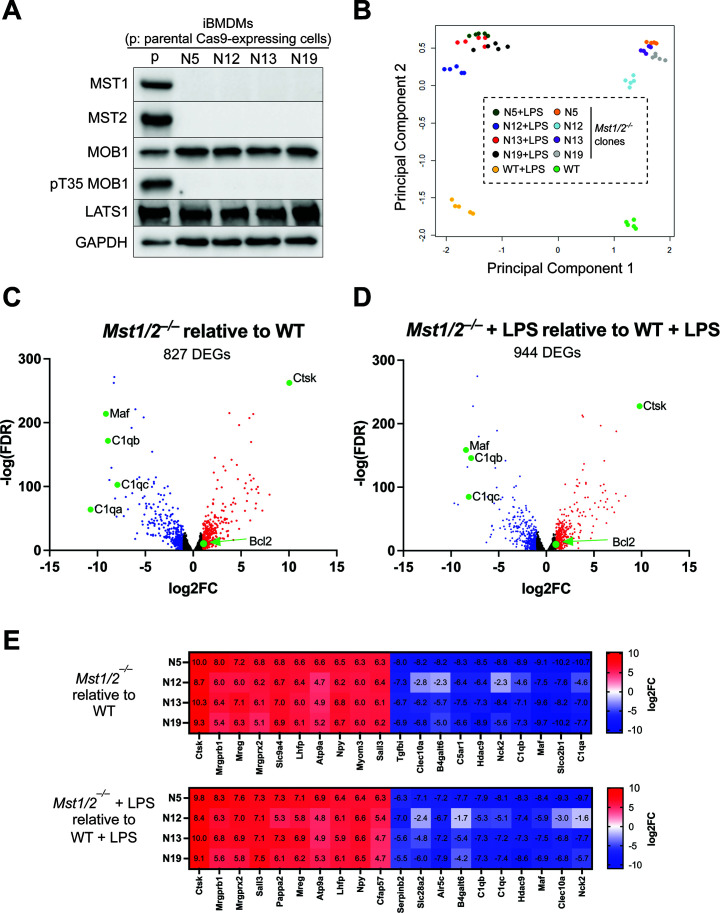
Global gene transcription controlled by MST1/2 in macrophages. (**A**) Loss of MST1/2 proteins and MOB1 phosphorylation in *Mst1/2^–/–^* iBMDMs. Cell lysates from the iBMDM clones were analyzed by immunoblotting for the indicated proteins and phosphorylation of the threonine 35 residue on MOB1 (pT35 MOB1). GADPH was used as an internal control. (**B**) Principal component analysis of gene expression profile similarities between WT iBMDMs and *Mst1/2^–/–^* clones treated with or without 2 µg/mL of LPS for 3 hours. Data represent samples collected from five independent repeats. (C and D) Volcano plots for gene expression of the representative *Mst1/2^–/–^* clone N5 compared to WT iBMDMs under the conditions of without LPS (**C**) and with LPS treatment (**D**) based on RNAseq data. DEGs were selected with greater than 100 transcript reads in either WT or *Mst1/2^–/–^* N5 and false discovery rate (FDR) < 0.01. Red, up-regulated DEGs with log2-fold change ≥1; blue, down-regulated DEGs with log2-fold change≤−1. Green spots labeled the genes were further analyzed or discussed in this study. (**E**) Expression heat map of the top 10 up- or down-DEGs in all four *Mst1/2^–/–^* clones without and with LPS treatment. The numbers within the color boxes indicate the log2-fold changes vs WT iBMDMs.

To investigate the role of the Hippo kinases in immune cells, we knocked out both *Mst1* and *Mst2* genes (*Mst1/2^–/–^*) in mouse immortalized bone marrow-derived macrophages (iBMDMs) using CRISPR-Cas9 genome-editing techniques. We obtained multiple *Mst1/2^–/–^* iBMDM clones (N5, N12, N13, and N19) that had no detectable levels of MST1 and MST2 (Fig. 1A). In these clones, the protein levels of LATS1 and MOB1 remained comparable to the levels in wildtype (WT) iBMDMs, but MOB1 phosphorylation was abolished since MOB1 is a known substrate of MST1/2, further confirming that MST1 and MST2 were absent in these cells (Fig. 1A). Next, we used RNA sequencing (RNAseq) to profile global gene expression in iBMDMs. WT and *Mst1/2^–/–^* iBMDMs were either treated with or without lipopolysaccharide (LPS), the outer membrane component of Gram-negative bacteria, to evaluate the effects of MST1/2 on macrophage gene expression under non-stimulated and stimulated conditions. The biological coefficient of variation plot showed that all four *Mst1/2^–/–^* clones clustered relatively close together and were markedly distinct from the WT macrophages (Fig. 1B). LPS treatment caused similar principal component shifts in WT and *Mst1/2^–/–^* macrophages (Fig. 1B). This global analysis showed that the *Mst1/2^–/–^* clones behave similarly, and MST1 and MST2 have significant impacts on macrophage gene expression.

Comparing the *Mst1/2^–/–^* N5 clone with the WT iBMDMs using these criteria, average reads >100 in N5 or WT; log2 fold-change >1 or <-1; false discovery rate (FDR) <0.01, 827 genes in the without LPS condition (*Mst1/2^–/–^* N5 vs WT) and 944 genes in the LPS stimulation condition (*Mst1/2^–/–^* N5 + LPS vs WT + LPS) were identified as differentially expressed genes (DEGs) (Fig. 1C and D; Table S1). Most of the top 10 up- or down-regulated genes had greater than 64-fold changes (log2-fold change >6 or <−6), suggesting that MST1 and MST2 are critical regulators for these genes (Fig. 1E). Many of the top DEGs identified in without LPS condition, for example *Ctsk*, *Maf*, *C1qb*, and *C1qc*, remained as the top DEGs in the condition with LPS, indicating that MST1 and MST2 directly regulate these top DEGs regardless of LPS treatment (Fig. 1C–E). Interestingly, none of the top DEGs were previously characterized as the signature genes regulated by YAP1 and WWTR1, the downstream transcription regulators in the canonical Hippo pathway (31). We further examined the expression levels of 22 YAP1/WWTR1 signature genes identified in cancers by the previous study (31). Surprisingly, the majority of the signature genes (17 out of 22) were poorly expressed in both WT and *Mst1/2^–/–^* iBMDMs (Fig. S1A), including *Ctgf* and *Cyr61* which are well-known marker genes controlled by YAP1/WWTR1. Among the five genes that had relatively high expression levels (average reads >100), only two genes, *Dock5* and *Myof*, were moderately up-regulated (log2 fold-change: 1.12–1.7) (Fig. S1B). The low abundance of the YAP1/WWTR1-controlled gene transcripts in iBMDMs is consistent in mouse primary macrophages, in which many of these YAP1/WWTR1 signature genes were also poorly transcribed when compared to other primary cell types in mice (Fig. S1C) (32–34). In addition, CTGF proteins were present in human embryonic kidney 293T cells but were not detectable in human THP1 macrophages by immunoblotting (Fig. S1D). Similarly, CTGF proteins were not detected in primary mouse macrophages and iBMDMs (Fig. S1D), indicating that immortalization does not appear to alter the expression of the YAP1/WWTR1 signature genes. Therefore, MST1 and MST2 possibly control gene expression through a non-canonical signaling cascade in macrophages.

### Pathways, biological processes, and molecular signatures controlled by MST1/2 in macrophages

To characterize the individual and combined interaction effects of *Mst1/2* gene knockouts and LPS stimulation, the gene lists were condensed and selected by more stringent criteria (see Materials and Methods), resulting in 360 significant genes for *Mst1/2^–/–^* vs WT iBMDMs, 706 significant genes shared by WT and *Mst1/2^–/–^* macrophages for LPS vs no LPS treatments, and 74 significant genes for combined interaction analysis. The significant genes were submitted to iPathwayGuide (Advaita Bioinformatics) for enrichment analyses, including pathways and gene ontology biological processes. The iPathwayGuide analyses confirmed that these macrophages were responsive to LPS stimulation since LPS was identified as the most significant upstream chemical identified from the LPS vs no LPS significant gene list (Fig. S2A). Marker genes of LPS stimulation, such as inducible NO synthase (*Nos2*), C-C motif chemokine ligand 5 (*Ccl5*), and interleukin 1α (*Il1a*), were highly induced in WT macrophages and all *Mst1/2^–/–^* clones (Fig. S2B). Volcano plots of WT +LPS vs WT (Fig. S2C) and *Mst1/2^–/–^*+LPS vs *Mst1/2^–/–^* (Fig. S2D) revealed similar induction of the LPS signature genes, suggesting that *Mst1/2^–/–^* macrophages have no defect in sensing and responding to LPS.

**Fig 2:**
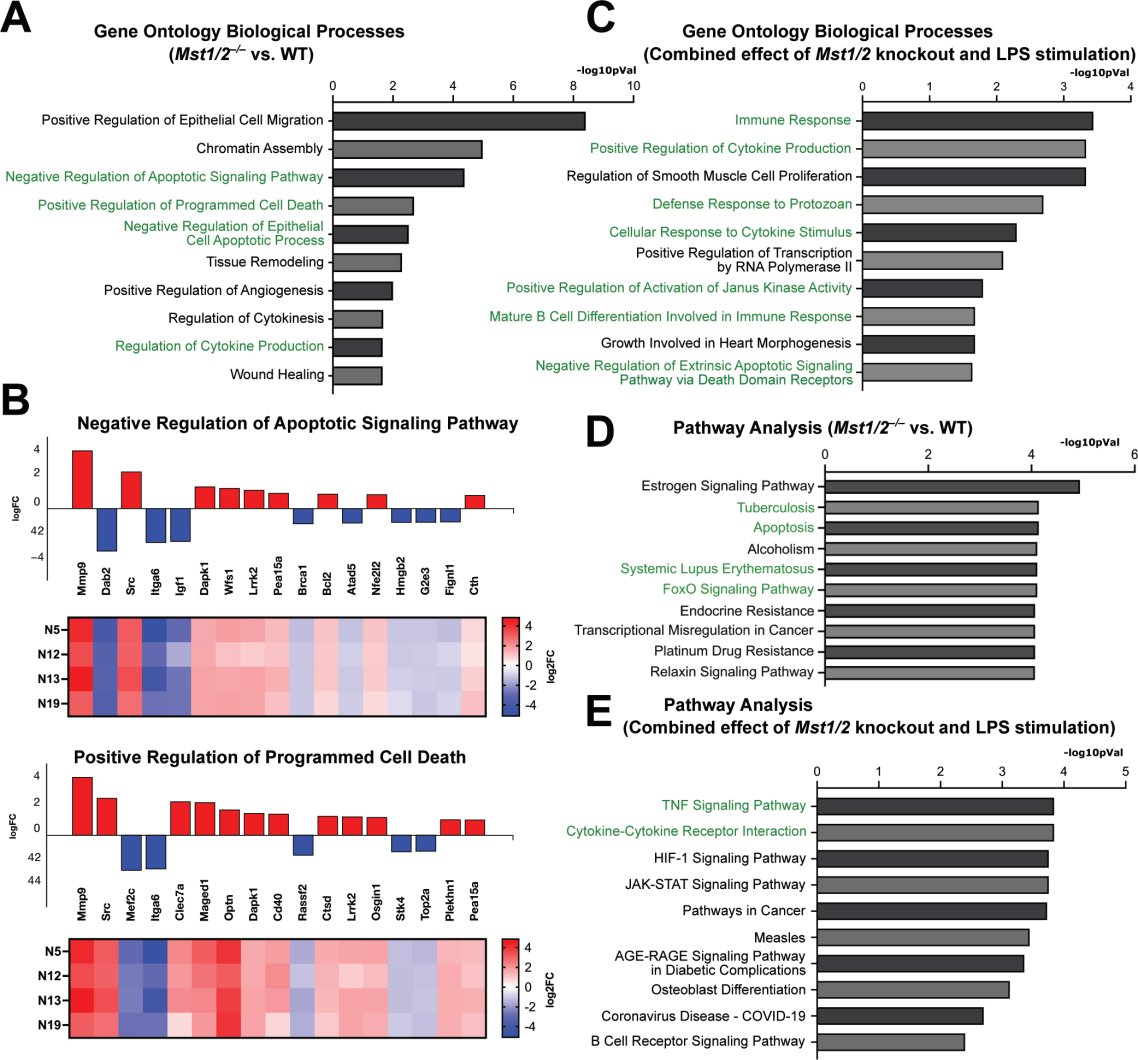
MST1 and MST2 are the key regulators of immune pathways and biological processes in macrophages. (**A)** Gene Ontology biological processes identified by the DEGs between WT and *Mst1/2^–/–^* iBMDMs. Up- and down-regulated significant gene profiles (360 significant genes) were combined between all *Mst1/2^–/–^* clones and provided to iPathwayGuide (AdvaitaBio). Processes are ranked by −log10 *P* value and smallest common denominator pruning is used for *P*-value correction. The top 10 processes with *P* values < 0.05 were presented. Processes or pathways involved in immunity or cell death are in green font. (**B)** Expression heat maps of four *Mst1/2^–/–^* clones for cell death-relevant processes identified in (**A**). Red, up-regulated; blue, down-regulated. (**C)** Gene Ontology biological processes identified from the 74 significant genes selected for the combined interaction effects of *Mst1/2* gene knockouts and LPS stimulation using iPathwayGuide. Processes were ranked by −log10 *P* value and smallest common denominator pruning is used for p-value correction. The top 10 processes with *P* values < 0.05 were presented. (**D)** KEGG pathways identified by iPathwayGuide based on the 360 significant genes of *Mst1/2^–/–^* vs WT iBMDMs. The FDRs were presented as −log10 *P* values and the top 10 pathways were presented. (**E) **KEGG pathways were identified by iPathwayGuide based on the 74 significant genes selected for the combined interaction effects of *Mst1/2* gene knockouts and LPS stimulation. The FDRs were presented as −log10 *P* values and the top 10 pathways were presented.

Next, we performed Gene Ontology (GO) analysis of the 360 significant genes from *Mst1/2^–/–^* vs WT iBMDMs and identified several processes involved in programmed cell death, such as apoptosis, and regulation of cytokine production among the top biological processes (Fig. 2A). Expression changes of genes involved in these processes were similar among the *Mst1/2^–/–^* clones (Fig. 2B). Analyzing the combined *Mst1/2* knockout and LPS interaction effect identified immune responses, cytokine production, and programmed cell death as the top GO biological processes (Fig. 2C). Likewise, the KEGG pathway analyses revealed that the genes from the *Mst1/2^–/–^* vs WT comparison were highly enriched in immune pathways and apoptosis (Fig. 2D). Notably, the identified pathways included the FoxO signaling pathway which has been shown in other cell types to be influenced by MST1/2 (35–37) (Fig. 2D). Consistent with the GO analyses, the TNF signaling pathway was identified as the top enriched pathway in the interaction effects of *Mst1/2* knockout and LPS treatment (Fig. 2E). Together, these bioinformatic results illustrate a role for MST1/2 in the regulation of gene expression related to a variety of inflammatory responses and programmed cell death in macrophages.

### MST1 and MST2 regulate cathepsin K (Ctsk) and B-cell lymphoma 2 (Bcl2) expression *via* non-canonical Hippo signaling

**Fig 3:**
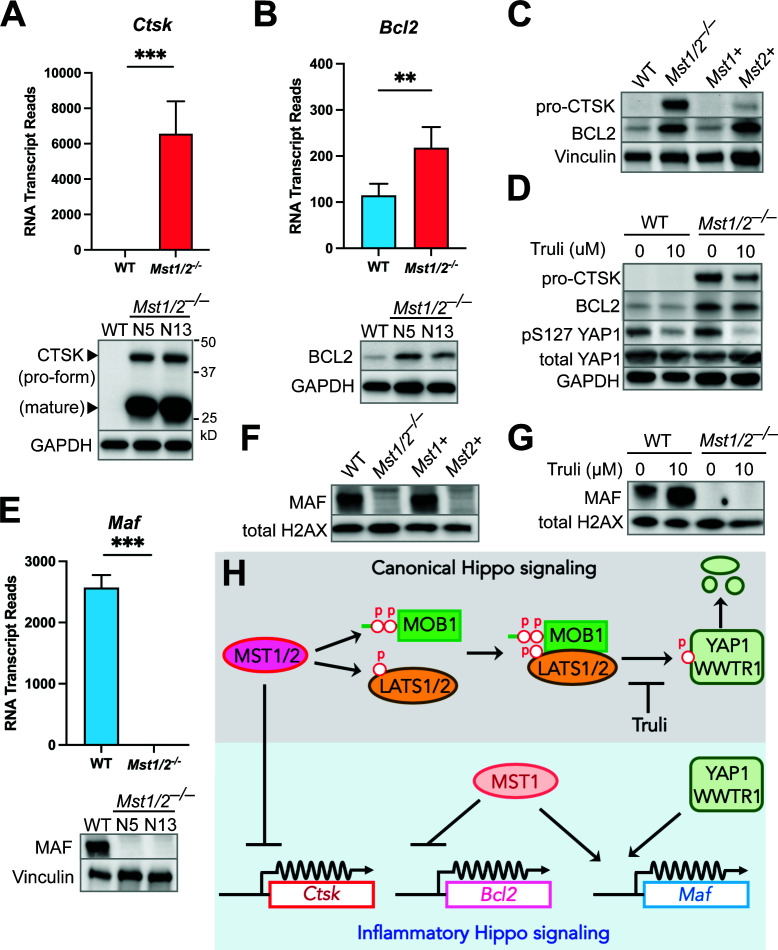
Inflammatory Hippo signaling for macrophage gene expression is independent of the canonical pathway. (A, B, and E) RNA and protein expression in WT and *Mst1/2^–/–^* iBMDMs. Top: Average RNA transcript reads for *Ctsk, Bcl2,* and *Maf* from the RNAseq data of *Mst1/2^–/–^* N5 clone and WT iBMDMs. RNA average reads from five independent repeats. Student’s *t*-test, two-tailed, unpaired, **: *P* < 0.01; ***: *P* < 0.001. Bottom: Protein levels of CTSK, BCL2, and MAF in the cell lysate of WT iBMDMs and two *Mst1/2^–/–^* iBMDM clones were detected by immunoblotting. (C and F) Levels of the indicated proteins in WT, *Mst1/2^–/–^*, MST1-expressing (*Mst1+*), and MST2-expressing (*Mst2+*) iBMDMs were determined by immunoblotting. (D and G) WT or *Mst1/2^–/–^* iBMDMs were treated with or without 10 µM Truli for 24 hours. Cells were then lysed, and protein samples were collected and analyzed by immunoblotting. Vinculin, GAPDH, and total histone H2AX were used as internal loading controls in immunoblots. All blots are representative of three independent repeats. (**H)** Schematic model of the regulatory activities of MST1 and MST2 through inflammatory Hippo signaling to control expression of *Ctsk*, *Bcl2,* and *Maf* in macrophages.

RNAseq revealed that *Ctsk* encoding the lysosomal protease cathepsin K was abundantly transcribed in *Mst1/2^–/–^* macrophages (Fig. 1C and Fig. 3A) but barely detected in WT macrophages. CTSK is highly expressed in osteoclasts, a type of macrophages involved in bone resorption, and plays a role in autoimmune disorders, such as psoriasis and systemic lupus erythematosus (38, 39). Since regulation of CTSK by MST1/2 was not reported before, we confirmed that both the pro-form and mature CTSK proteins were highly produced in *Mst1/2^–/–^* macrophages but not detectable in WT macrophages by immunoblotting (Fig. 3A). Given that BCL2 is a well-characterized regulator of apoptosis (40) and one of the DEGs controlled by MST1/2 (Fig. 1C), we also determined BCL2 protein expression. Consistent with the levels of RNA transcripts, increased BCL2 protein levels were detected in *Mst1/2^–/–^* macrophages (Fig. 3B). MST1 and MST2 are highly similar (Fig. S3) and both can phosphorylate the downstream Hippo components, such as MOB1 (6). To investigate whether MST1 and MST2 have shared or distinct activities in gene expression, we generated iBMDMs expressing either MST1 (*Mst1+*) or MST2 (*Mst2+*) only. While CTSK was highly expressed in the double-knockout *Mst1/2^–/–^* macrophages, its protein level was suppressed in *Mst1+* or *Mst2+* macrophages (Fig. 3C). *Mst1+* macrophages, like WT macrophages, had low levels of BCL2, but *Mst2+* macrophages had increased BCL2 proteins comparable to the level in *Mst1/2^–/–^* macrophages (Fig. 3C), revealing that the two kinases have overlapping but also separate activities in gene expression.

When canonical Hippo signaling is on, MST1 and MST2 phosphorylate and activate the downstream kinases LATS1/2 which phosphorylate YAP1/WWTR1 (Fig. 3H). Since CTSK and BCL2 expression were increased in *Mst1/2^–/–^* macrophages, we used a nucleotide-analog inhibitor, Truli (41), to specifically block the kinase activity of LATS1/2, resulting in an off status of canonical Hippo signaling that is similar to knocking out *Mst1/2*. As anticipated, Truli reduced YAP1 phosphorylation on the serine residue 127 (Fig. 3D). No change in BCL2 protein expression was detected in WT or *Mst1/2^–/–^* macrophages treated with Truli (Fig. 3D). Truli also did not induce CTSK expression in WT macrophages and, unexpectedly, caused a moderate decrease in CTSK in *Mst1/2^–/–^* macrophages (Fig. 3D), indicating that the expression of CTSK and BCL2 is controlled by MST1/2 *via* alternative routes other than canonical Hippo signaling.

### MST1 up-regulates MAF expression through a mechanism that is not canonical Hippo signaling

We next characterized the expression of *Maf*, one of the highly down-regulated genes in *Mst1/2^–/–^* macrophages (Fig. 1C). MAF is a leucine zipper transcription factor (42) and controls key functions in macrophages, including self-renewal, cell death, and cytokine production (43–46). While both RNA and protein expression of *Maf* were detected in WT macrophages, lack of MST1/2 resulted in no detectable MAF (Fig. 3E). Unlike CTSK being negatively controlled by both MST1 and MST2, only *Mst1+* macrophages restored MAF expression (Fig. 3F). Interestingly, the Truli treatment led to an increase in MAF expression in WT macrophages while MAF remained undetectable in *Mst1/2^–/–^* macrophages (Fig. 3G). This result reveals a unique regulatory loop that the presence of MST1 kinase positively regulates MAF expression, likely through a separate mechanism, and canonical Hippo signaling mediated by LATS1/2 kinases suppresses MAF expression. Together with CTSK and BCL2 regulation, these findings show that MST1 and MST2 have shared and distinct activities in macrophage gene expression *via* signaling cascades independent of canonical Hippo signaling (Fig. 3H).

### MST1 and MST2 affect the release of cytokines and chemokines in macrophages

**Fig 4:**
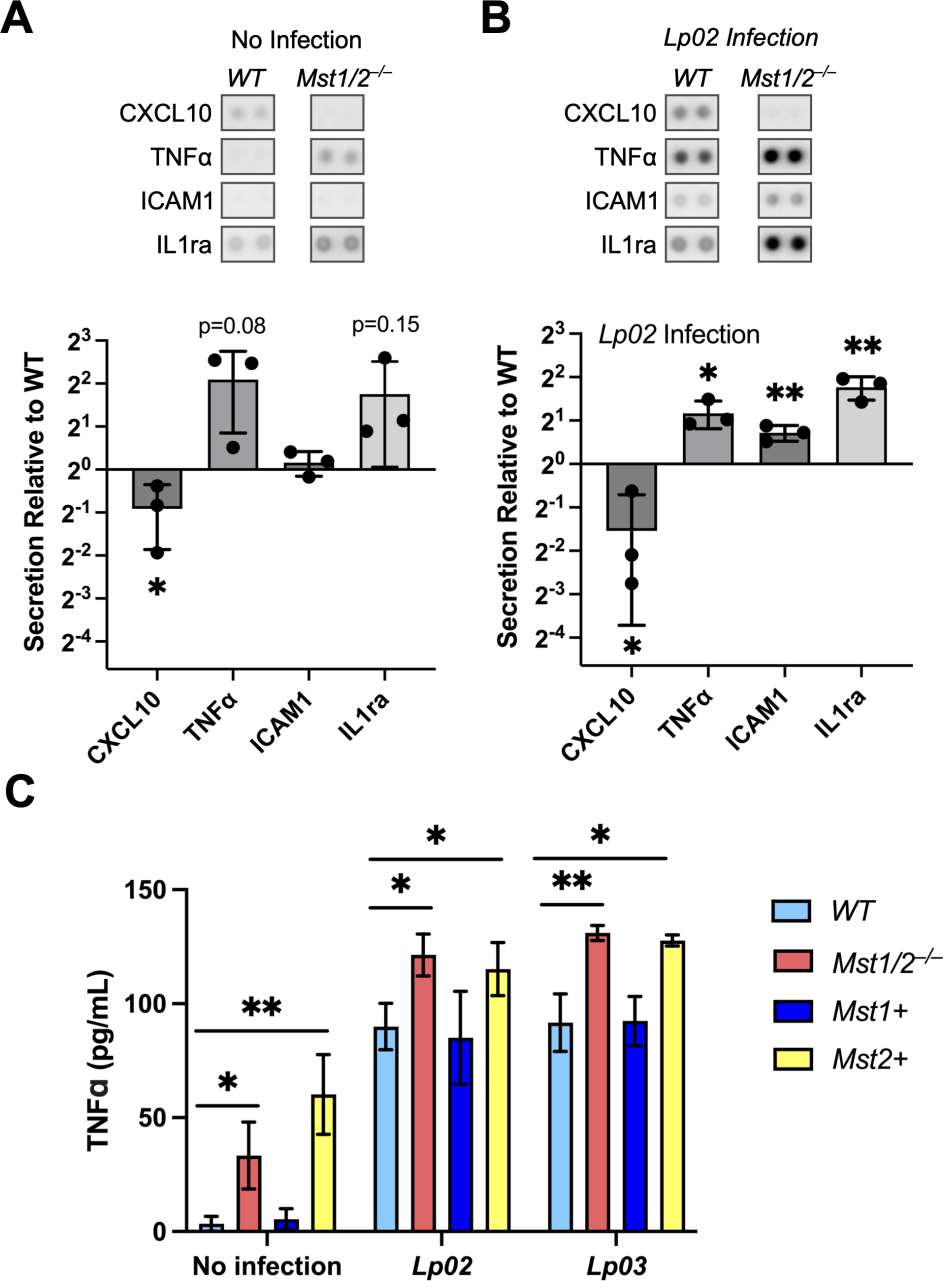
MST1 and MST2 affect post-translational cytokine release in macrophages. (A and B) The presence of cytokines and chemokines in the conditioned media collected from WT and *Mst1/2^–/–^* iBMDMs over 3 hours of incubation without infection (**A**) or with challenge of the *L. pneumophila Lp02* strain at a multiplicity of infection (MOI) 10 (**B**) was detected by the cytokine arrays. Representative images of the cytokine spots on the arrays were presented. Graphs show relative secretion levels of the indicated cytokines in *Mst1/2^–/–^* and WT iBMDMs. The intensity of the cytokine spots was quantified using ImageJ and normalized to the intensity of the corresponding spots of WT iBMDMs (set as 1). Data were plotted as log2-fold change and presented as mean ± SD of three independent cytokine array experiments. (**C)**. Quantitation of TNFα secretion in WT, *Mst1/2^–/–^*, *Mst1+,* and *Mst2+* BMDMs by ELISA. Conditioned media were collected from iBMDMs with or without challenge of the *L. pneumophila* strains (MOI = 10) over 3 hours of incubation. Concentrations of TNFα in the conditioned media were presented as mean ± SD (pg/mL) of three independent experiments. Student’s *t*-test, two-tailed, unpaired, *P < 0.05; **P < 0.01.

GO analyses identified several biological processes involved in cytokine production were potentially affected by MST1/2 (Fig. 2). Therefore, we used cytokine arrays to detect the presence of 40 cytokines in the conditioned media collected from WT and *Mst1/2^–/–^* macrophages over 3 hours of incubation (Fig. S4A). The relative secretion level of CxC motif chemokine 10 (CXCL10) was significantly reduced in the conditioned media of *Mst1/2^–/–^* macrophages (Fig. 4A) while secretion of TNFα and interleukin-1 receptor antagonist protein (IL1ra) was enhanced but not statistically significant. We challenged the iBMDMs with the Gram-negative bacterial pathogen *Legionella pneumophila* to determine whether MST1 and MST2 affect host cytokine release during infection. Upon challenge of the virulent *L. pneumophila* strain *Lp02* for 3 hours, the relative levels of TNFα, IL-1ra, and intercellular adhesion molecule 1 (ICAM1) were significantly higher in the conditioned media collected from *Mst1/2^–/–^* macrophages than from WT macrophages (Fig. 4B). The level of CXCL10 secretion remained significantly reduced in *Mst1/2^–/–^* macrophages challenged with *Lp02* (Fig. 4B). The results from the cytokine arrays show that MST1 and MST2 have a role in secretion of a variety of cytokines and chemokines by macrophages.

To determine whether MST1 and MST2 have different effects on cytokine secretion, we measured TNFα release by the *Mst1/2* double- and single-knockout iBMDMs using quantitative enzyme-linked immunosorbent assays (ELISA). TNFα is a multifunctional cytokine that mediates cell-to-cell communication and modulates immune responses, including activation of nuclear factor κB for cell survival and the extrinsic apoptosis pathway (47–50). Confirming the cytokine array results, over ninefold higher concentrations of TNFα were detected in the conditioned media of *Mst1/2^–/–^* macrophages than WT macrophages (average 33.3 pg/mL in *Mst1/2^–/–^* vs 3.5 pg/mL in WT; Fig. 4C). *Mst1+* and WT macrophages released low levels of TNFα. Notably, *Mst2+* macrophages released more TNFα than WT and *Mst1+* macrophages at a level that was slightly and non-significantly higher than the level of *Mst1/2^–/–^* macrophages (Fig. 4C). Similar results were observed in conditioned media collected from the cells over 24 hours of incubation (Fig. S4B). We then compared TNFα release by the macrophages challenged with the virulent *L. pneumophila Lp02* strain or a non-virulent *Lp03* strain that lacks the type IV secretion system (T4SS). Challenging with either *Lp02* or *Lp03* stimulated the release of TNFα by the macrophages. The TNFα levels in *Mst1/2^–/–^* and *Mst2+* macrophages remained significantly higher than in WT or *Mst1+* macrophages although the levels among these cells seemed to be relatively comparable (Fig. 4C). Importantly, induction of TNFα release is not a result of lytic cell death. While the conditioned medium from WT macrophages challenged with the virulent *Lp02* strain contained increased levels of the cytosolic enzyme lactate dehydrogenase (LDH) as an indicator of lytic cell death, the non-virulent *Lp03* strain did not cause cell lysis (Fig. S4C) but induced TNFα release to the same level as the *Lp02* strain did (Fig. 4C). Consistently, the increased TNFα release observed in *Mst1/2^–/–^* and *Mst2+* macrophages under the no infection condition was not associated with spontaneous cell death since all four lines of macrophages showed similar levels of background LDH release (Fig. S4D). Together, these results suggest that MST1 plays a suppressive role in TNFα secretion and the role of MST2 could be minor.

### MST1 and MST2 promote apoptotic cell death in macrophages

**Fig 5:**
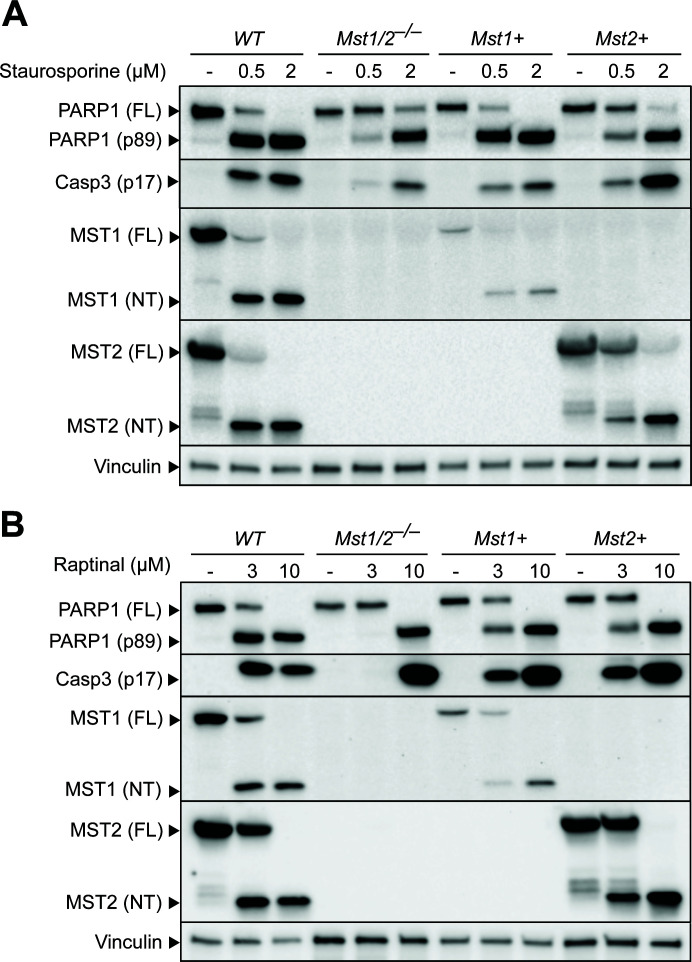
MST1 and MST2 control activation of apoptosis in macrophages. Wildtype, *Mst1/2^–/–^, Mst1+,* and *Mst2+* iBMDMs were treated with either staurosporine for 4 hours (**A**) or Raptinal for 3 hours (**B**) to induce apoptosis. Cell lysates were collected and levels of PARP1, MST1, MST2, activated caspase-3 (**p17**), and vinculin (as an internal control) were detected by immunoblotting.

Since the role of MST1/2 in macrophage cell death was undetermined, we tested the activation of apoptosis in the iBMDMs with *Mst1/2* double- or single-knocked out. Treating WT macrophages with the apoptosis-inducing agent, staurosporine, triggered the cleavage of poly-ADP-ribose polymerase-1 (PARP1) to an 89 kD fragment, a hallmark of apoptosis, and production of the activated apoptosis executioner caspase-3 (Casp3, p17) (Fig. 5A). In addition, we detected cleavage of full-length (FL) MST1/2 into the MST1/2 N-terminal (MST1/2-NT) fragments, a phenomenon that is also observed in several cell lines treated with staurosporine or Fas ligands (12–14, 51). In comparison to staurosporine which is a broad-spectrum kinase inhibitor, we treated the macrophages with Raptinal, a recently discovered agent that induces fast apoptosis *via* the intrinsic mitochondria pathway (52, 53). Raptinal induced production of MST1/2-NT and triggered apoptosis in WT macrophages (Fig. 5B). Importantly, when *Mst1/2^–/–^* macrophages were treated with low doses of staurosporine or Raptinal, PARP1 cleavage and Casp3 activation were reduced or minimal (Fig. 5). *Mst1+* or *Mst2+* macrophages triggered cleavage of MST1 or MST2, respectively, and had levels of apoptosis comparable to the level in WT iBMDMs, suggesting that MST1 or MST2 alone is sufficient to activate apoptosis induced by these treatments (Fig. 5). At high doses of staurosporine (2 µM) or Raptinal (10 µM), *Mst1/2^–/–^* macrophages underwent apoptosis (Fig. 5), indicating that excessive stimuli can bypass the requirement of MST1/2 to induce apoptosis. Overall, these findings demonstrate that MST1 and MST2 are important modulators of the apoptotic death pathway in macrophages.

### MST1 and MST2 are the key factors in macrophages to restrict bacterial infection

**Fig 6:**
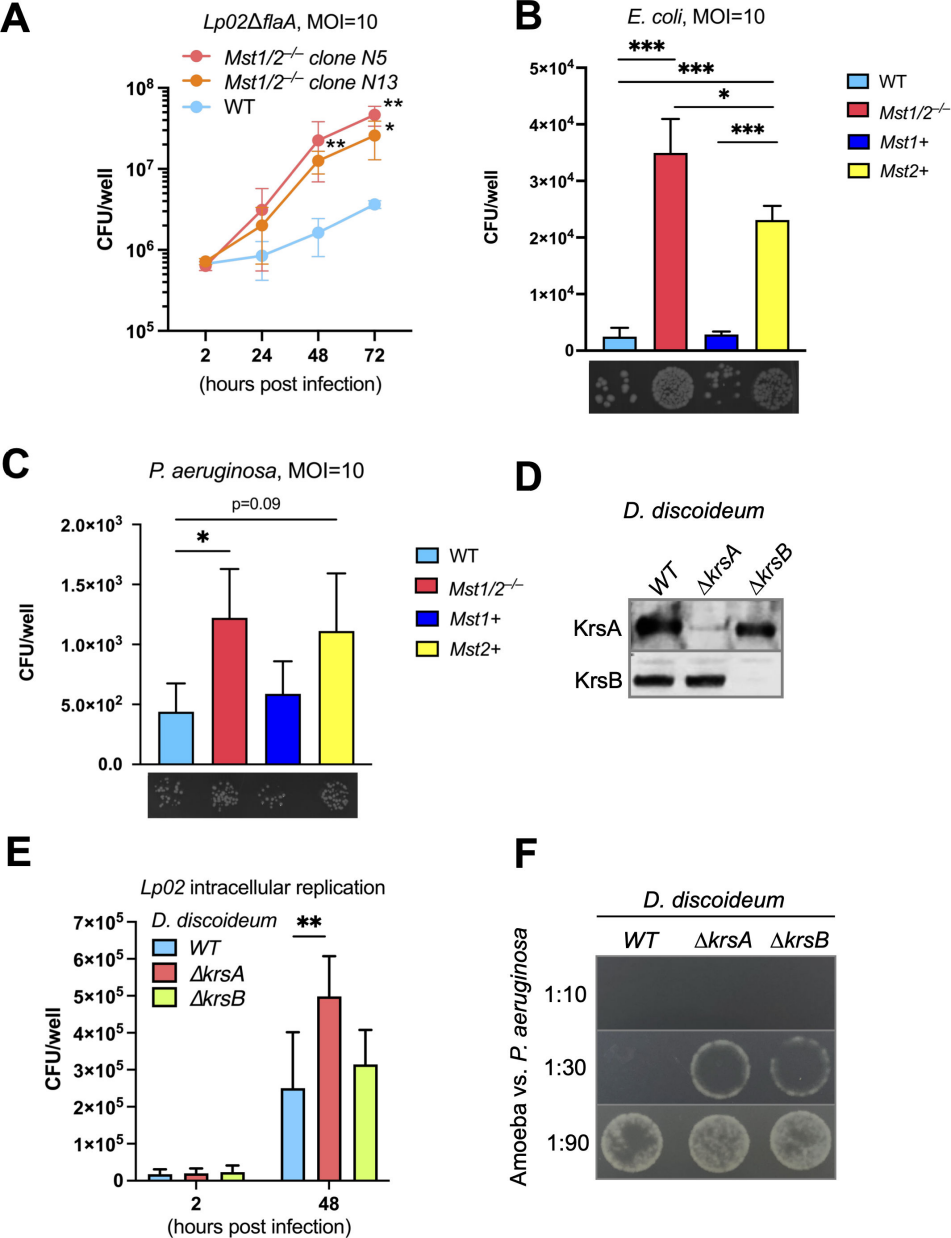
The conserved Hippo kinases in eukaryotic hosts restrict bacteria. (**A**) WT and *Mst1/2^–/–^* iBMDMs were challenged with *L. pneumophila ∆flaA* at MOI = 10. After 2 hours, free bacteria were removed by washing. At the indicated post-infection time points, iBMDMs were lysed with digitonin to release intracellular bacteria. The numbers of *L. pneumophila* in the cell lysate were determined by the colony forming units (CFUs) assay. Data presented as mean ± SD of three independent experiments. Student’s *t*-test, two-tailed, unpaired, *P < 0.05; **P < 0.01 compared to WT. (B and C**)** iBMDMs were infected with *E. coli* or *P. aeruginosa* at MOI = 10. After 1 hour, gentamicin (200 µg/mL) was added to kill extracellular bacteria. The infected iBMDMs were incubated for an additional 2 hours and then lysed with Triton X-100. The cell lysate was serially diluted and plated on LB agar plates to count bacterial CFUs. Data presented as mean ± SD of three independent experiments. Images represent the formation of bacterial colonies on the plates. (**D**) Protein levels of the Hippo kinases KrsA and KrsB in the *D. discoideum* strains were determined by immunoblotting. (**E) ***D. discoideum* amoebae were challenged with the *L. pneumophila Lp02* strain at MOI = 10. The numbers of *L. pneumophila* at the indicated post-infection time points were determined by the CFU assay as in (**A**). Data presented as mean ± SD of six independent experiments. (**F)** The ability of the *D. discoideum* strains to prey on bacteria was determined by the semi-quantitation of resistance to predation. The amoebae were mixed with *P. aeruginosa* at the indicated ratios and the mixture was spot-plated on SM5 agar plates. Surviving bacteria grew on the plates after overnight incubation at 22℃. Images are representative of two independent experiments. Statistic method: Student’s *t*-test, two-tailed, unpaired, *P < 0.05; **P < 0.01.

Given that humans with MST1 deficiency experience recurrent infection (19, 20), we sought to determine whether a lack of MST1/2 reduces macrophages’ ability to restrict invading bacteria. We challenged WT and *Mst1/2^–/–^* iBMDMs with a virulent *L. pneumophila* strain *Lp02ΔflaA* which had the flagellin gene deleted and measured its intracellular replication. The *Lp02∆flaA* strain was used to avoid interference from macrophage pyroptosis, a lytic form of programmed cell death, triggered by the presence of flagellin. Over the 72-hour infection period, *Lp02∆flaA* had moderate intracellular growth in WT macrophages but grew robustly in *Mst1/2^–/–^* macrophages (Fig. 6A), suggesting that MST1 and MST2 are required to restrict replication of *L. pneumophila*. To further elucidate the individual role of MST1/2 in restricting bacterial infection, we challenged WT, *Mst1/2^–/–^*, *Mst1+,* or *Mst2+* iBMDMs with *Escherichia coli* and performed a gentamicin protection assay. *Mst1/2^–/–^* macrophages had severe defects in restricting *E. coli* (Fig. 6B). Interestingly, *Mst2+* macrophages had increased susceptibility to *E. coli* although the level was significantly lower than the level in *Mst1/2^–/–^* macrophages (Fig. 6B). Expression of MST1 was sufficient to suppress *E. coli* infection. Similar results were obtained from the iBMDMs challenged with another bacterial pathogen *Pseudomonas aeruginosa* using the gentamicin protection assay (Fig. 6C). These results suggest that MST1 appears to play a major role in the restriction of the bacteria and MST2 may have mild anti-bacterial activities.

### The Hippo kinases are conserved restriction factors against bacteria in protozoan hosts

*L. pneumophila* and *P. aeruginosa* are both opportunistic human pathogens and exist in natural environments. The Hippo pathway is highly conserved in eukaryotes, including free-living protozoans that constantly encounter and prey on bacteria. We further investigated whether the Hippo kinase homologs, KrsA and KrsB (54) (Fig. S5), of the soil amoeba *Dictyostelium discoideum* affect the interactions with the bacteria. We obtained *D. discoideum* strains with *krsA* or *krsB* gene deletion and confirmed loss of KrsA or KrsB proteins in these strains (Fig. 6D). The *D. discoideum* strains were challenged with the *L. pneumophila* strain *Lp02* since *D. discoideum* cells do not undergo pyroptosis like macrophages. Because of the virulence factor T4SS, the *Lp02* strain was capable of replicating within *D. discoideum* and increased intracellular replication of *L. pneumophila* was observed in the *∆krsA* amoebae (Fig. 6E). We then used a semi-quantitative assay of resistance to predation to measure the ability of the *D. discoideum* strains in preying on and killing *P. aeruginosa*. In this assay, the amoebae and bacteria were mixed at different ratios and spotted on the SM5 agar plates to allow bacteria predation by amoebae. If amoebae failed to ingest and kill bacteria, surviving bacteria grew on the plates (55). As shown in Fig. 6F, the *∆krsA* and *∆krsB* strains were defective in predation or killing of *P. aeruginosa*. Therefore, the Hippo kinases also play a critical role in host defense in amoebae.

## DISCUSSION

The Hippo kinases MST1/2 phosphorylate MOB1 and LATS1/2 to modulate the transcriptional activity of YAP1/WWTR1, which is well known as the canonical Hippo pathway (2, 3). Here, we investigate the diverse activities of the Hippo kinases in gene expression, release of cytokines/chemokines, programmed cell death, and antimicrobial defense in macrophages. Intriguingly, while being homologous proteins, MST1 and MST2 share certain functions but also individually possess unique activities. For example, both MST1/2 control macrophage apoptosis but have different effects on TNFα cytokine release (Fig. 5 and 4C). Moreover, we demonstrate that the Hippo kinases control anti-microbial activities in macrophages and amoebae, reflecting a conserved role of these kinases in determining host susceptibility to infection.

Since the data sets available for the transcriptome controlled by MST1/2 are mainly collected in non-immune cells, we profiled global gene expression of the macrophages lacking both MST1 and MST2. The MST1/2 transcriptome reveals genes that were previously not known to be regulated by MST1/2, such as *CtsK*, *Bcl2,* and *Maf*, implying that MST1 and MST2 control macrophage gene expression *via* an alternative signaling cascade (Fig. 1 and 3). This is also supported by the fact that most of the YAP1/WWTR1 target genes in non-immune cells are poorly expressed in macrophages (Fig. S1). Indeed, if CTSK and BCL2 expression were increased due to shutting off canonical Hippo signaling in *Mst1/2^–/–^* macrophages, blocking the activity of LATS1/2 by Truli would result in increased expression of CTSK or BCL2 in WT macrophages. The Truli treatment does not affect CTSK or BCL2 expression (Fig. 3D). Likewise, the Truli treatment promotes MAF expression in WT macrophages, an effect opposite to the suppressed expression observed in *Mst1/2^–/–^* macrophages (Fig. 3G). Taken together, MST1 and MST2 regulate these representative genes in macrophages by alternative signaling cascades which we term the inflammatory Hippo pathway to distinguish from the canonical Hippo pathway (Fig. 3H).

The transcriptome analyses indicate the importance of MST1/2 in immune pathways (Fig. 2). The cytokine arrays and ELISAs confirm the effects of MST1/2 on the release of several cytokines and chemokines (Fig. 4). Although the effects of alteration in cytokine/chemokine release on immune cell communication remain to be determined, these results indicate that post-translational protein trafficking is under control of the Hippo kinases in macrophages. In addition, MST1 and MST2 have different activities in regulating TNFα release by macrophages (Fig. 4C). Challenging with *L. pneumophila* markedly increased TNFα release in all the macrophage clones, and similar levels of TNFα were detected between macrophages challenged with the virulent T4SS-expressing *Lp02* strain and the non-virulent T4SS-null *Lp03* strain, suggesting that *L. pneumophila*-induced TNFα release is independent of the virulence factor T4SS and lytic cell death (Fig. 4C; Fig. S4C). Similar effects on TNFα release have been observed in macrophages challenged with live or heat-killed *L. pneumophila* (56). Thus, MST1, but not MST2, has a role in preventing premature TNFα release in macrophages prior to encountering bacteria.

Both MST1 and MST2 can phosphorylate MOB1 and LATS1/2 to activate the canonical Hippo pathway (Fig. 3H). Expression of either MST1 or MST2 markedly reduces CTSK expression (Fig. 3C), although the results from the Truli experiment suggest that the suppression is mediated by an alternative, non-canonical Hippo signaling. On the other hand, like their different roles in TNFα release, we observed that MST1 has significant impacts on BCL2 and MAF expression while MST2 has no effect (Fig. 3C and F). The differences between MST1 and MST2 in gene regulation further demonstrate that these Hippo kinases affect immunity independent of the canonical Hippo pathway. Indeed, MST1/2 can affect autophagy by phosphorylating LC3, and the LATS1/2 kinases can be activated by an alternative route through MAP4Ks without MST1/2 (57–59).

Our results reveal the implications of the inflammatory Hippo pathway in macrophage differentiation and autoimmune diseases. For example, MST1 is essential for the expression of MAF which has a role in cell death, inflammatory responses of tumor-associated macrophages, and macrophage differentiation (44–46, 60). MST1 and MST2 strongly suppress the expression of CTSK that is highly produced in osteoclasts, the bone macrophages, and have been a therapeutic target for treating osteoporosis (61). CTSK also contributes to the pathogenesis of systemic lupus erythematosus (SLE) (38), one of the top pathways identified in the bioinformatic analyses (Fig. 2C). This is correlated with the expression of the complement genes *C1qa, b, c* being highly suppressed in *Mst1/2^–/–^* macrophages (Fig. 1C and D). Although the *C1q* genes were not selected for the iPathwayGuide analysis due to the stringent criteria used, C1q deficiency is a pathogenic factor of SLE (62, 63). Collectively, identifying MST1/2 as key regulators of these genes offers insights to manage anti-tumor immunity or mitigate osteoporosis and SLE.

Programmed cell death is an important host defense in innate immunity (64). The pro-apoptotic activity of MST1/2 has been characterized in cancer cell lines or fibroblasts by overproducing the kinases or gene knockdown methods (12–16). By contrast, *Mst1/2^–/–^* naïve T cells are prone to apoptosis (18–20). We found that MST1 and MST2 are both required for macrophage apoptosis with low-dose treatments of staurosporine and Raptinal but can be bypassed if the cells are over-stimulated at high doses (Fig. 5), indicating that macrophages with *Mst1/2* knockouts still possess the essential components of the apoptotic death pathway and the gene knockouts did not cause secondary mutations in the apoptotic cascade. BCL2 has anti-apoptosis activity by preventing activation of the intrinsic pathway. While *Mst1/2^–/–^* and *Mst2+* macrophages express increased BCL2 (Fig. 3C), *Mst2+* macrophages still undergo apoptosis when treated with the apoptosis agents at low doses (Fig. 5), suggesting that the resistance to apoptosis in *Mst1/2^–/–^* macrophages is likely caused by other mechanisms. The C-termini of MST1 and MST2 have self-inhibitory activity, and removal of the C-termini by proteases increases the kinase activity of MST1/2, resulting in activation of apoptosis (65). Therefore, MST1/2-NT produced upon the treatments may lead to apoptosis in macrophages (Fig. 5). Notably, studies showed that the apoptotic caspase Casp3 cleaves MST1/2 and produces MST1/2-NT to promote apoptosis in non-macrophage cell lines (12, 14). In this study, *Mst1/2* knockout macrophages have reduced Casp3 activation in response to the low-dose treatments (Fig. 5). These observations suggest that MST1/2 can regulate Casp3 activation and function as modulators of apoptosis in macrophages.

The results from the infection experiments (Fig. 6) demonstrate the critical role of MST1/2 in anti-bacterial host defense, showing a correlation with the increased susceptibility to infection observed in humans with MST1 deficiency and *Mst1/2* double knockout mice (19, 20, 23). *Mst1/2^–/–^* macrophages fail to limit infections by *E. coli*, *P. aeruginosa,* and *L. pneumophila* (Fig. 6A to C). We found that MST1 has a more pivotal role in anti-bacterial activities than MST2 (Fig. 6B and C) and that MST1/2 may activate different host defense mechanisms against different bacteria. For example, expression of MST2 in macrophages partially restores the anti-bacterial activity against *E. coli* but not *P. aeruginosa* (Fig. 6B and C). Similarly, *∆krsA* amoebae are more permissive to the intracellular bacterium *L. pneumophila* while either *∆krsA* or *∆krsB* amoebae are incompetent in preying on and killing the extracellular bacterium *P. aeruginosa* (Fig. 6E and F). This indicates that the single Hippo kinase knockout *D. discoideum* also has different types of defects in host defense. Collectively, the Hippo kinases are key factors in controlling a range of host defense mechanisms in mammalian macrophages and protozoans depending upon the types of pathogen challenge. Future studies that dissect these mechanisms in host cells at molecular levels are necessary to understand the central role of these conserved Hippo kinases in host-pathogen interactions and innate immunity.

## MATERIALS AND METHODS

### Mammalian cell culture

Mouse iBMDMs were gifts from Dr. Jonathan Kagan. Cells were cultured in Dulbecco’s Modified Eagle’s medium (DMEM, Corning 15013CV) supplemented with 10% fetal bovine serum (FBS, Corning 35–016-CV), 2 mM L-glutamine (Corning, MT25005CI), and 0.1% penicillin/streptomycin (*P*/S, Corning, MT3000CI) at 37°C in humidified incubators with 5% CO_2_. Cells were passaged every 2–3 days when they reached 60%–80% confluency. Human embryonic kidney 293T (HEK293T, ATCC, CRL3216) cells were cultured in DMEM supplemented with 10% FBS, 2 mM L-glutamine, and 0.1% penicillin/streptomycin. Human THP1 macrophages (ATCC, TIB202) were cultured in RPMI 1640 (ATCC modification, Gibco A1049101) supplemented with 10% FBS and differentiated with 100 nM phorbol 12-myristate 13-acetate (PMA, Sigma-Aldrich, P8139) for 24 hours. Cell lysate of primary mouse bone marrow-derived macrophages was a gift from the laboratory of Dr. Yuan He at Wayne State University (66).

### Knocking out Mst1 and Mst2 genes in iBMDMs

LentiCas9-Blast (Addgene #52962) plasmid containing human codon-optimized *Streptococcus pyogenes* Cas9 protein and *Stk3* (*Mst2*, Addgene #75975, gift from John Doench & David Root (67)) gRNA was used to dually target *Mst1* and *Mst2*, as the gRNA sequence is identical in both genes and packaged in lentiviral particles. Lentiviral particles were generated by co-transfection with the packaging plasmids pMDLg/RRE (Addgene #12251), pMD2.G (Addgene #12259), and pRSV-Rev (Addgene #12253) into HEK293T/17 cells for 24 hours. The packaging plasmids were obtained from Addgene and gifts of Didier Trono (68). Following transfection, culture supernatant was collected, centrifuged, and filtered. Culture supernatants were added to parental iBMDMs to undergo a 72-hour transduction. Following the transduction, iBMDMs were selected using 10 µg/mL blasticidin. For *Mst1* and *Mst2* double or single knockouts, Cas9-expressing iBMDMs were transduced for 72 hours with viral particles containing *Stk3* gRNA. Following transduction, iBMDMs were selected using 10 µg/mL blasticidin and 10 µg/mL puromycin. Blasticidin- and puromycin-resistant iBMDMs were isolated through limited dilutions and amplification. Cells were then lysed using 1× Laemmli sample buffer and proteins were analyzed by immunoblotting to examine MST1 and MST2 expression. Cas9-expressing iBMDMs were used as the wildtype control in all following experiments.

### Bacterial culture

*E. coli* strain MG1655 was suspended in 2.5 mL of regular LB broth (10 g/L tryptone, 5 g/L yeast extract, and 10 g/L NaCl) and incubated overnight at 37°C in a shaking incubator. *Pseudomonas aeruginosa* strain PAO1F was suspended in 2.5 mL liquid high salt LB broth (10 g/L tryptone, 5 g/L yeast extract, 11.7 g/L NaCl, 10 mM MgCl_2_, and 0.5 mM CaCl_2_) and incubated overnight at 37°C in a shaking incubator. The next day, the bacterial cultures were diluted at 1:50 or 1:100 in fresh LB broth (for *E. coli*) or high salt LB (for *P. aeruginosa*) and incubated for 2–3 hours at 37°C in a shaking incubator. The fresh bacterial culture was pelleted at 11,000 × *g* for 2 minutes. Bacterial pellets were resuspended in DMEM without FBS or antibiotics. The density of bacterial resuspension was measured at optical density (OD_600_) and used to calculate the dilutions for the desired multiplicity of infection (MOI).

*Legionella pneumophila* carrying the plasmid pJB908 was cultured on CAYE agar plate (2 g/L activated charcoal, 10 g/L ACES, 10 g/L yeast extract, 400 µg/mL cysteine, 135 µg/mL ferric nitrate, 50 µg/mL streptomycin, and 15 g/L agar), and *L. pneumophila* patches were resuspended in liquid AYE broth (10 g/L ACES, 10 g/L yeast extract, 400 µg/mL cysteine, and 135 µg/mL ferric nitrate) and incubated at 37°C. Overnight cultures were centrifuged at 11,000 × *g* for 2 minutes, then resuspended in DMEM supplemented with 10% FBS and 2 mM L-glutamine without antibiotics. The density of bacterial resuspension was measured at optical density (OD_600_) and used to calculate the dilutions for the desired MOI.

### Dictyostelium discoideum culture

*D. discoideum*, including the wildtype AX2 parental strain (strain ID: DBS0350762), *∆krsA* strain (strain ID: DBS0350759), and *∆krsB* strain (strain ID: DBS0350760), were obtained from the DictyBase stock center (Northwestern University, Chicago, USA). Axenic *D. discoideum* cells were cultured in HL-5 media (14 g/L glucose, 7 g/L yeast extract, 14 g/L thiotone, 0.95 g/L Na_2_HPO_4_-7H_2_O, 0.5 g/L KH_2_PO_4_, and pH 6.5) at 21°C in a microbiological incubator. The amoeba cells were sub-cultured every 2 days. For measuring *L. pneumophila* intracellular replication, the amoebae were harvested and resuspended in MB media (3.9 g/L MES, 7 g/L yeast extract, 14 g/L thiotone, and pH 6.9). 5 × 10^5^ *D. discoideum*/well were seeded in 24-well plates and incubated at 26°C for 2 hours. 50 µL of *L. pneumophila* suspension in MB media was added into the wells to challenge the *D. discoideum* at MOI = 10. The plates were centrifuged at 200 × *g* for 5 minutes and incubated at 26°C for additional 2 hours. After the incubation, the wells were washed with fresh MB media three times to remove extracellular *L. pneumophila*. 0.5 mL/well of fresh MB media was added into the wells and the plates were incubated at 26°C in a microbiological incubator. At the desired post-infection timepoints, infected *D. discoideum* cells were lysed with sodium deoxycholate (final concentration 0.05%) to release intracellular *L. pneumophila*. The lysate was serially diluted with sterile water and spot-plated on CAYE agar plates, and the numbers of *L. pneumophila* were determined by colony forming units (CFUs) assays.

### Gentamicin protection assays

iBMDMs (1 × 10^5^ cells/well) were seeded into 24-well plates. The next day, the media was removed and replaced with fresh DMEM without FBS or P/S. iBMDMs were challenged with *E. coli* or *P. aeruginosa* and centrifuged at 200 × *g* for 5 minutes to facilitate contact between bacteria and iBMDMs. Cells were incubated at 37°C for 1 hour followed by adding 200 µg/mL gentamicin. Cells were incubated for an additional 2 hours, then washed three times with DMEM and lysed with 500 µL of 0.1% Triton-X100 in sterile water per well for 10 minutes. The lysate was serially diluted and spotted onto LB agar plates. Plates were incubated overnight at 30°C and colonies were counted the following day to determine colony forming units (CFUs).

### Semi-quantitation assays of resistance to predation of *D. discoideum*

*D. discoideum* cells cultured in MB media were harvested and resuspended in SorC buffer (2 g/L KH_2_PO_4_, 0.55 g Na_2_HPO_4_-7H_2_O, 50 µM CaCl_2_) at 5 × 10^6^, 1.675 × 10^6^, and 0.56 × 10^6^ cells/mL (threefold serial dilutions). Overnight *P. aeruginosa* cultures were 1:100 diluted in high salt LB and cultured at 37°C for 2–3 hours to reach the mid-log growth phase. *P. aeruginosa* was pelleted and resuspended in SorC buffer at 5 × 10^7^ bacteria/mL. 100 µL (5 × 10^6^) of *P. aeruginosa* was mixed with 100 µL of the serially diluted *D. discoideum* to have the bacteria: amoeba ratios at 10:1, 30:1, and 90:1. The amoeba/bacteria mixtures were spot-plated on SM5 agar plates (2 g/L glucose, 2 g/L bactopeptone, 2 g/mL yeast extract, 0.2 g/L MgSO_4_-7H_2_O, 1.9 g/L KH_2_PO_4_, 1 g/L K_2_HPO_4_, and 20 g/L agar). The SM5 agar plates were incubated at 22°C in a microbiological incubator and imaged the next day.

### *L. pneumophila* intracellular replication in iBMDMs

WT and *Mst1/2^–/–^* iBMDMs were seeded at 1 × 10^5^ cells/well in 24-well plates one day prior to infection. *L. pneumophila* with an in-frame deletion of the flagellin gene *flaA* (*Lp02∆flaA*) overnight cultures were pelleted and resuspended in fresh DMEM without P/S. The culture media in the 24-well plates were replaced with 900 µL of fresh DMEM without P/S, and 100 µL of *L. pneumophila* suspension was added to the wells to challenge the macrophages at MOI = 10. The plates were centrifuged at 200 × *g* for 5 minutes and incubated in the cell culture incubator. After 2 hours of incubation, the wells were washed with 1× PBS three times to remove free bacteria, and 1 mL of fresh DMEM without P/S was added. The plates were incubated in a cell culture incubator to the indicated post-infection timepoints. To count the numbers of *L. pneumophila* at the indicated time points, iBMDMs were lysed with 0.01% digitonin to release intracellular *L. pneumophila*. The lysate was serially diluted with sterile water and spot-plated on CAYE agar plates. The agar plates were incubated at 37°C in a microbiological incubator for 4 days and the colony forming units were calculated.

### RNA sequencing

WT and *Mst1/2^–/–^* iBMDMs were seeded in 6-well plates at 3 × 10^5^ cells/well. After culturing for 48 hours, the media was removed and replaced with fresh DMEM with or without LPS (2 µg/mL) for 3 hours. Total RNA samples from five independent repeats were extracted using a Qiagen RNAeasy mini kit (Cat. #74104) according to the manufacturer’s manual. RNA samples were sent to the Genomics Core at Wayne State University for sequencing and analyses. RNA quality control and quantification were performed on the TapeStation 4100, and libraries were prepared with 100 ng input using the QuantSeq 3′ mRNA-seq Library Prep Kit. Prepared RNA libraries were sequenced on the NovaSeq 6000 (>10M reads per sample). Reads were aligned to the mouse genome (mm10) with STAR for alignment followed by ht-seq count for tabulation for each gene region. Differential expression was analyzed with the log2-fold change between conditions, *P*-value, false discovery rate corrected *P*-value, and individual read counts.

### Pathway and Gene Ontology biological process analyses

RNA sequencing read counts were transformed by multiplying by 1,000,000 and dividing by the total number of reads for each sample to obtain counts per million reads mapped (CPM), which was then log2 transformed (after adding a small positive constant) to account for the inherent skewness of RNAseq count data. For each measured gene, the optimal statistical model describing the relationship between *Mst1/2* double knockout status and LPS treatment with CPM was chosen using the Bayesian Information Criterion. These investigated models included main effect only and interaction models. Bioinformatics analyses were then conducted using iPathwayGuide (Advaita) including and excluding interaction effects with a reduced gene pool of those with a ± log2-fold change, *P*-value < 0.05, and an overall mean of at least two on the CPM scale.

### Primary cell transcriptome data collection and analysis from FANTOM5

The Gene Expression Atlas (EMBL-EBI; https://www.ebi.ac.uk/gxa/home) was queried for the 22 YAP1/WWTR signature genes in mice (*Mus musculus*). Query was sorted by primary cell type and transcripts per million (TPM) for each gene were collected from the RIKEN FANTOM5 project of RNA sequencing Cap Analysis of Gene Expression (CAGE) (32–34). TPMs for five different primary cells (hepatic sinusoidal endothelial cell, fibroblast, astrocyte, cardiac muscle cell, and macrophage) were downloaded and graphed using GraphPad Prism.

### Cytokine arrays and ELISAs

iBMDMs were seeded in 12-well plates (3 × 10^5^/well). The following day, the media was removed and replaced with 1 mL of fresh DMEM, and the cells were incubated at 37°C for 3 or 24 hours. The culture media were centrifuged at 200 × *g* for 5 minutes and 800 µL of supernatant was collected. The supernatant was centrifuged again at 2,000 × *g* for 10 minutes to remove cell debris. The supernatant after the second centrifugation was analyzed by the cytokine array kits (R&D systems, ARY006) or the TNFα ELISA kit (BioLegend, #430904) according to the procedures described in the manufacturers’ manuals. Cytokine array analyses were performed by quantitation of spot intensity using ImageJ.

### Colorimetric cytotoxicity assay for LDH Release

Release of the cytosolic enzyme LDH into conditioned media was determined by a CytoTox 96 non-radioactive cytotoxicity assay (Promega, G1780) based on the manufacturer’s protocol. iBMDMs were challenged with *L. pneumophila* at MOI = 25 or uninfected for 3 hours. Supernatants were collected and transferred to a 96-well plate. LDH substrate was added to each well and incubated at room temperature for 20 to 30 minutes. After incubation, stop solution was added to each well and absorbance at 490 nm was measured using a SpectraMax i3X plate reader (Molecular Devices). All samples were analyzed in triplicate and normalized as percentages of the total cell lysate-positive control (iBMDMs treated with 0.02% Triton-X100). Data were presented as mean ± S.D. of three independent biological repeats.

### Truli, Raptinal, and staurosporine treatment

iBMDMs (3 × 10^5^ cells/well) were seeded one day prior to experimentation in 12-well plates. Prior to the treatments, media was removed and replaced with fresh DMEM. iBMDMs were treated with DMSO vehicle control, Truli (N-(3-Benzylthiazol-2(3H)-ylidene)−1H-pyrrolo[2,3-b] pyridine-3- carboxamide; CSN pharm CSN26140) or Raptinal (Adipogene, AG-CR1-2902) and incubated in CO_2_ humidified 37°C incubators for 3 hours. For staurosporine experiments, iBMDMs were treated with DMSO vehicle control or staurosporine (Cayman chemical, 81590) for 4 hours.

### Immunoblotting

Following the treatments, cell culture media was transferred to microcentrifuge tubes and centrifuged 200 × *g* for 5 minutes to collect floating cells. During centrifugation, cell monolayers in 12-well plates were lysed with 1X Laemmli buffer. Following centrifugation, the supernatant was removed, and the remaining cell pellet was combined with respective cell lysate from the treatments. Cell lysate was denatured at 97°C for 10 minutes and analyzed by SDS-PAGE and immunoblotting. Immunoblots were developed by horse-radish peroxidase (HRP) chemiluminescence and imaged using the BioRad Chemidoc Imaging system. Antibodies used: vinculin (sc-73614), GAPDH (sc-25778), BCL2 (sc-7382), CTGF (sc-365970), and CTSK (sc-48353) from Santa Cruz Biotechnology; MST2 (ab52641) from Abcam; MST1 (14946S), cleaved caspase-3 (9661S), PARP1 (9542S), phospho-S139 H2AX (2577S), total H2AX (2595S), MOB1 (13730S), phospho-T35 MOB1 (8699S), and LATS1 (3477S) from Cell Signaling; MAF (A300-613A) from Bethyl Laboratories; Rabbit polyclonal antibodies against *D. discoideum* KrsA and KrsB were gifts from Dr. Yulia Artemenko (State University of New York, Oswego) (54). All immunoblots are representative of at least three biological repeats.

### Statistical analyses and graphing

Statistical analyses and graphs were performed and produced in GraphPad Prism.

## Data Availability

RNA sequencing raw data are deposited in NIH Sequence Read Archive (SRA) and can be accessed via the BioProject accession numbers: PRJNA1052713, PRJNA1052715, PRJNA1052722, PRJNA1052731, PRJNA1052734. Other data are presented in this manuscript or available in the supplementary materials.

## SUPPLEMENTAL MATERIAL

Figure S1 mbio.03429-23-s0001.eps Detection of YAP1/WWTR1-regulated genes in macrophages by RNA sequencing.

Figure S2 mbio.03429-23-s0002.eps WT and *Mst1*/*2*^-/-^ iBMDMs respond to LPS treatment.

Figure S3 mbio.03429-23-s0003.eps Amino acid sequence alignments of MST1 and MST2.

Figure S4 mbio.03429-23-s0004.eps MST1/2 regulate secretion of cytokines into the conditioned media.

Figure S5 mbio.03429-23-s0005.eps Amino acid sequence alignments of the Hippo kinases in mouse, human, and *D. discoideum* amoeba.

Legends mbio.03429-23-s0006.docx Supplemental figure legends.

Caption mbio.03429-23-s0007.docx Table S1 caption.

Table S1 mbio.03429-23-s0008.xlsx Differentially expressed genes in WT or *Mst1*/*2* double knockout clone N5 iBMDMs with or without LPS stimulation.

ASM does not own the copyrights to Supplemental Material that may be linked to, or accessed through, an article. The authors have granted ASM a non-exclusive, world-wide license to publish the Supplemental Material files. Please contact the corresponding author directly for reuse.
