# Supplementary material for: The Hippo kinases control inflammatory Hippo signaling and restrict bacterial infection in phagocytes
Source: mBio. 2024 Apr 16;15(5):e03429-23. doi: 10.1128/mbio.03429-23 (PMC11078001; doi:10.1128/mbio.03429-23)
Supplement: Legends — Supplemental figure legends. [file mbio.03429-23-s0006.docx]

**Figure S1. Detection of YAP1/WWTR1-regulated genes in macrophages by RNA sequencing. A.** Average transcript reads in iBMDMs by RNAseq for 22 established YAP1/WWTR1-regulated genes based on Wang et al., 2018 (reference 31). The five genes with average reads >100 are highlighted in yellow boxes. **B.** Heat expression maps for the five YAP1/WWTR1-regulated genes with average reads > 100 in *Mst1/2^–/–^* iBMDMs. The values within the boxes indicate the log2-fold changes of gene expression compared to WT iBMDMs. **C.** Average Transcripts per Million (TPM) for 22 established YAP1/WWTR1-regulated genes in primary cell types from FANTOM5 gene expression atlas (reference 32-34). **D.** Levels of CTGF protein in various human and mouse cell types was determined by immunoblotting. Total histone H2AX is used as an internal control.

**Figure S2. WT and *Mst1/2^–/–^* iBMDMs respond to LPS treatment*.* A.** 706 differentially regulated genes by lipopolysaccharides (LPS) treatment in WT and *Mst1/2^–/–^* iBMDMs were provided to iPathwayGuide. Upstream chemicals were predicted by iPathwayGuide and ranked by -log10 p value. This analysis confirms that iPathwayGuide accurately predicted LPS as the inducer for the differentially regulated genes in iBMDMs. **B.** Expression heat maps for the top 12 up-regulated genes in WT and *Mst1/2^–/–^* iBMDMs by LPS treatment. Similar levels of expression induction by LPS treatment were observed in WT and *Mst1/2^–/–^* iBMDMs. **C and D.** Volcano plots for the gene expression of LPS-treated WT iBMDMs vs. untreated WT iBMDMs (**C**) and LPS-treated *Mst1/2^–/–^* N5 iBMDMs vs untreated *Mst1/2^–/–^* N5 iBMDMs (**D**) based on RNAseq data. DEGs were selected with greater than 100 transcript reads and false discovery rate (FDR) < 0.01. Red, up-regulated DEGs; blue, down-regulated DEGs. Green spots indicate the top 12 up-regulated genes in WT and *Mst1/2^–/–^* iBMDMs with LPS treatment.

**Figure S3. Amino acid sequence alignments of MST1 and MST2*.* A.** Comparisons of amino acid sequences of mouse MST1 (NCBI Protein ID: NP_067395) and MST2 (NCBI Protein ID: NP_062609). Schematic diagrams generated by Clustal Omega (EMBL-EBI) and ESPript 3 (SBGrid). Identical amino acids are in the red background and white font. Similar amino acids are in the white background and red font. Numbers indicate the positions of amino acid residues of MST1.

**Figure S4. MST1/2 regulate secretion of cytokines into the conditioned media. A.** Representative images of the cytokine arrays (R&D Systems) that detect 40 cytokines and chemokines in the conditioned media collected from WT and *Mst1/2^–/–^* iBMDMs challenged with *Lp02* for 3 hours. Array images shown are representative of three independent biological repeats. Cytokine or chemokine spots are manufactured in duplicate on the arrays, and the color boxes define the spots of cytokines or chemokines showing differential levels of secretion. **B.** WT, *Mst1/2^–/–^*, *Mst1+*, and *Mst2+* iBMDMs were cultured for 24 hours and the conditioned media was collected. ELISAs were used to determine the concentration of TNFα in culture media between groups. Data shown is representative of four biological repeats. **C.** WT iBMDMs were challenged with the *L. pneumophila* strains for 3 hours and LDH release into conditioned media was quantified by a colorimetric cytotoxicity assay and presented as percentages of total LDH in culture media of iBMDMs lysed with Triton-X100. Data were presented as mean±SD of three independent experiments. **D.** LDH release in conditioned media collected from WT, *Mst1/2^–/–^, Mst1+, Mst2+* iBMDMs without infection over 3 hours incubation was determined and presented as described in (**C**). LDH release data in (**C**) and (**D**) are as mean ± SD (pg/mL) of three independent experiments. Student’s t-test, two tailed, unpaired, **: p <0.01. No statistical significance detected among the iBMDMs clones in (**D**).

**Figure S5. Amino acid sequence alignments of the Hippo kinases in mouse, human and *D. discoideum* amoeba.** Comparisons of amino acid sequences among the Hippo kinases from *Mus musculus* (mMST1 NCBI Protein ID: NP_067395; mMST2 NCBI Protein ID: NP_062609), *Homo sapiens* (hMST1, NCBI Protein ID: NP_006273; hMST2, NCBI Protein ID: NP_006272) and *Dictyostelium discoideum* (dKrsA, NCBI Protein ID: XP_638650; dKrsB, NCBI Protein ID: XP_647461)*.* Schematic diagrams generated by Clustal Omega (EMBL-EBI) and ESPript 3 (SBGrid). Identical amino acids are in the red background and white font. Similar amino acids are in the white background and red font. Numbers indicate the positions of amino acid residues of mMST1. We note that full-length KrsB has 1,105 amino acids and the N-terminal 526 residues with sequence homology to the other Hippo kinases are shown in the alignments.
