# Supplementary material for: The Hippo kinases control inflammatory Hippo signaling and restrict bacterial infection in phagocytes
Source: mBio. 2024 Apr 16;15(5):e03429-23. doi: 10.1128/mbio.03429-23 (PMC11078001; doi:10.1128/mbio.03429-23)
Supplement: Caption — Table S1 caption. [file mbio.03429-23-s0007.docx]

**Supplemental Table 1**. Lists of differentially expressed genes in WT or *Mst1/2* double knockout clone N5 iBMDMs with or without LPS stimulation. The genes were selected by these criteria: average RNA transcript reads in either N5 or WT iBMDMs>100, log2-fold change>1 or <-1, false discovery rate (FDR)<0.01. 827 differentially expressed genes were selected in no LPS condition (N5 vs. WT), and 944 genes were selected in the LPS stimulation condition (N5+LPS vs. WT+LPS). 1,145 differentially expressed genes were selected in WT+LPS vs. WT condition and 1,269 genes were selected in N5+LPS vs N5 condition.
